# The proportion of impervious surfaces at the landscape scale structures wild bee assemblages in a densely populated region

**DOI:** 10.1002/ece3.2374

**Published:** 2016-08-25

**Authors:** Benoît Geslin, Violette Le Féon, Morgane Folschweiller, Floriane Flacher, David Carmignac, Eric Motard, Samuel Perret, Isabelle Dajoz

**Affiliations:** ^1^ CNRS, IRDInstitut Méditerranéen de Biodiversité et d'Ecologie Marine et Continentale (IMBE) Aix Marseille UniversitéAvignon Université Pôle St Jérôme av. Escadrille N. Niemen13397Marseille Cedex 20France; ^2^ CNRS, UMR 7618iEES‐ParisF‐75005ParisFrance; ^3^ INRA, UMR 406 Abeilles et EnvironnementINRASite AgroparcF‐84914Avignon Cedex 9France; ^4^ Université ParisDiderot‐7F‐75013ParisFrance; ^5^ Université Pierre et Marie Curie‐6F‐75005ParisFrance; ^6^ CEREEP Ecotron ÎleDeFranceUMS CNRS 3194Saint‐Pierre‐lès‐NemoursFrance

**Keywords:** Bees, community ecology, gradient, landscape scale, urbanization

## Abstract

Given the predicted expansion of cities throughout the world, understanding the effect of urbanization on bee fauna is a major issue for the conservation of bees. The aim of this study was to understand how urbanization affects wild bee assemblages along a gradient of impervious surfaces and to determine the influence of landscape composition and floral resource availability on these assemblages. We chose 12 sites with a proportion of impervious surfaces (soil covered by parking, roads, and buildings) ranging from 0.06% to 64.31% within a 500 m radius. We collected using pan trapping and estimated the landscape composition of the sites within a 500 m radius and the species richness of plant assemblages within a 200 m radius. We collected 1104 bees from 74 species. The proportion of impervious surfaces at the landscape scale had a negative effect on wild bee abundance and species richness, whereas local flower composition had no effect. Ground‐nesting bees were particularly sensitive to the urbanization gradient. This study provides new evidences of the impact of urbanization on bee assemblages and the proportion of impervious surfaces at the landscape scale emerged as a key factor that drives those assemblages.

## Introduction

One of the major causes of the current bee decline is the destruction of natural habitats (Brown and Paxton 2009; Winfree et al. 2009; Goulson et al. 2015) due to agricultural intensification and increasing urbanization (Steffan‐Dewenter et al. 2002; Tscharntke et al. 2005; McKinney 2006, 2008; Winfree et al. 2009). Urbanization permanently alters habitats and destroys natural areas that include floral resources and nesting sites for wild bees (McKinney 2002; Banaszak‐Cibicka and Żmihorski 2012). This impact is likely to increase in the near future due to the predicted expansion of cities worldwide (McDonnell and Hahs 2008; Hennig and Ghazoul 2011a). Thus, urbanization and its impact on bees and pollinators in general have received increasing attention over the past few years (see Hernandez et al. 2009 for a review; Bates et al. 2011; Bergerot et al. 2011; Matteson et al. 2012; Geslin et al. 2013; Fortel et al. 2014; Verboven et al. 2014). In urban environments, the main factor affecting pollinators appears to be the amount of impervious surfaces at the landscape scale with related impacts of habitat loss and fragmentation (Ahrné et al. 2009; Banaszak‐Cibicka and Żmihorski 2012; Geslin et al. 2013). However, although many authors have reported negative effects of urbanization on bee assemblages (Hernandez et al. 2009; Bates et al. 2011), other studies have argued that cities might support relatively high levels of bee abundance and/or species richness (McIntyre et al. 2001; Fetridge et al. 2008; Matteson et al. 2008; Fortel et al. 2014), particularly when local management strategies promote green spaces (e.g., parks and seminatural remnants) and abundant floral resources (Wojcik and McBride 2011; Matteson et al. 2012). In general, urbanization appears to act as a filter for bee communities by promoting large‐bodied and aboveground‐nesting species and inhibiting small‐bodied and ground‐nesting bees in urban matrices (Banaszak‐Cibicka and Żmihorski 2012; Geslin et al. 2013). Despite an increase in the number of studies examining the link between bees and urbanization in the past few year (*e.g.,* Sattler et al. 2010a,b; Bates et al. 2011; Fortel et al. 2014; Threlfall et al. 2015), our knowledge of the effect of urbanization on bee assemblages is still incomplete. In particular, we need to acquire more information to disentangle the respective effects of local management practices and the degree of urbanization at the landscape scale on bee assemblages.

To our knowledge, the majority of studies have considered an urbanization gradient from the first agglomeration belt around relatively small cities to their centers (e.g., Stockholm in Ahrné et al. 2009; Birmingham in Bates et al. 2011 and Poznań in Banaszak‐Cibicka and Żmihorski 2012). As reported by Lin and Fuller (2013), regional‐scale studies of the impact of urbanization on biodiversity are urgently needed. Here, we chose a regional‐scale urbanization gradient in the most densely populated part of France, the Île‐de‐France region (an area of 12,000 km^2^ surrounding Paris, France). This area is acknowledged as a very densely populated area worldwide (Pereira et al. 2013), and it encompasses a great diversity of habitats, such as seminatural, agricultural, suburban, and densely urbanized landscapes. In this region, the urbanization continues to increase primarily at the cost of agricultural areas (Torre et al. 2013). We selected 12 sites that formed a gradient with respect to impervious surfaces within a 500 m radius as a proxy for the degree of urbanization. Impervious surface coverage has emerged as a key environmental factor to describe urbanization over the past several years (Arnold and Gibbons 1996). This indicator is widely used in studies of the effects of urbanization on bees (Ahrné et al. 2009; Banaszak‐Cibicka and Zmihorski, 2012; Geslin et al. 2013; Fortel et al. 2014), as well as on other taxa (e.g., wasps in Zanette et al. 2005 and plants in Pellissier et al. 2012). In ecology, it expresses the proportion of an area covered by buildings, parking areas, pavements, and roads (Marzluff 2005; Sattler et al. 2010a,b; Liu et al. 2014). Moreover, an increase in the proportion of impervious surfaces often implies joint modifications of the ecosystems at landscape and local scales (in addition to habitat loss in terms of floral resources and nesting sites) such as an increase in the ambient temperature, a soil compaction, and also soil and air pollution (McKinney 2002). In all twelve sites, we analyzed the influence of landscape composition and the structure of the local plant assemblage on bee assemblages. The latter was taken into account because the structure of bee assemblages could be strongly linked to the structure of local plant assemblages (Potts et al. 2003).

Our aims were twofold: (1) to understand how urbanization affects wild bee abundance, species richness, and assemblage composition and (2) to determine the influence of landscape composition and floral resource availability on bee assemblages. Answering these questions is essential for the development of management strategies that will promote sustainable bee communities.

## Methods

### Study sites

**Figure 1:**
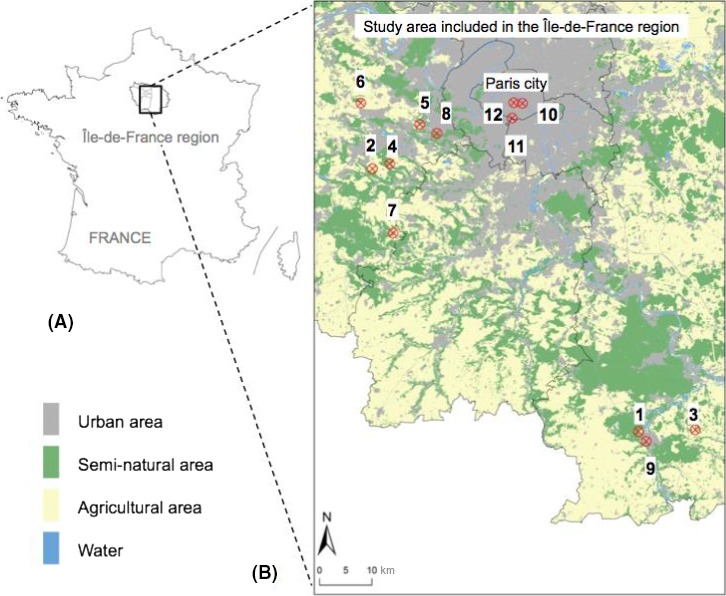
Location of the study area in France and in the Île‐de‐France region (black box) (A) and location of the 12 study sites (with site number) in the study area (B).

Our study system was located in the administrative region of Paris (Ile‐de‐France), which is the most densely populated region of France (more than 11 million inhabitants, INSEE 2013). Twelve sites situated at least at 1 km from each other (min = 1.66 km; max = 89.31 km; mean ± SE = 38.31 ± 28.17 km) were selected according to their proportion of impervious surfaces within a 500 m radius to cover an urbanization gradient (Fig. 1). The effect of landscape composition on bee assemblages has been previously studied at larger scales, that is, 1 km (Blaauw and Isaacs 2014; Hopfenmüller et al. 2014) up to 3 km (Steffan‐Dewenter et al. 2002; Westphal et al. 2003). We chose a 500 m radius because it encompasses the estimated mean flight distances of the majority of wild bees species (Gathmann and Tscharntke 2002; Araújo et al. 2004; Franzén et al. 2009; Zurbuchen et al. 2010; Wright et al. 2015). Furthermore, it is widely used in studies linking bee assemblages and landscape composition (Holzschuh et al., 2008; Somme et al. 2014), especially within urbanization contexts (Ahrné et al. 2009; Banaszak‐Cibicka and Żmihorski 2012; Geslin et al. 2013; Fortel et al. 2014).

The proportion of impervious surfaces ranged from 0.06 to 64.31% (mean ± SE = 25.25 ± 24.58%). In addition to this urbanization gradient, the land cover composition of the twelve sites also reflected the diversity of habitats in the Île‐de‐France region, with sites dominated by crops, forests, or grasslands (Table 1). We used Geographic Information Systems (ArcGisV.10.0, Redlands, CA, USA) and French Corine Land Cover data (Bossard et al. 2006) to determine the proportion of the following eight land cover categories: permanent grasslands, forests, crops, private gardens, public gardens, bare ground, impervious surfaces (buildings, parking lots, pavements, and roads), and water‐covered surfaces. Bare ground was included because of its importance as a habitat for ground‐nesting bees (Michener 2007). Private and public gardens were considered separately. Both have been shown to offer suitable living conditions for wildlife (see Gaston et al. 2005; Muratet and Fontaine 2015; Sattler et al. 2010a,b for private gardens and Pawelek et al. 2009; Gunnarsson and Federsel 2014 for public gardens), but management practices may be different in public parks with policies aiming at reducing pesticide use (Pawelek et al. 2009; Muratet and Fontaine 2015). In Paris, in particularly, managers of public parks have been encouraged to reduce their impact on wildlife through biodiversity‐friendly management (Shwartz et al. 2013). Crops largely consisted of wheat and corn, but mass flowering crops (oilseed rape, alfalfa, and peas) were also present in small proportions (5–15%) at sites 2, 3, 5, and 6. We also calculated the total proportion of seminatural areas by pooling the proportions of forests and permanent grasslands. Three sites (sites 1, 4, and 5) hosted a percentage of seminatural areas superior to 50%, and three sites (2, 3, and 6) presented a proportion of crops superior to 40%. Three sites (7, 8, and 9) were located in small cities around Paris and showed a percentage of impervious surfaces between 25% and 50%. Finally, three sites (10, 11, 12) were located in Paris city and had a percentage of impervious surfaces above 50% (Table 1).

**Table 1:** Description of sites land cover (in %). Seminatural areas represent the proportion of grasslands and the proportion of forests pooled

	Site1	Site2	Site3	Site4	Site5	Site6	Site7	Site8	Site9	Site10	Site11	Site12
Impervious surfaces	0.061	0.5	1.33	5.55	7.01	9.33	27.38	33.09	40.57	53.78	60.12	64.31
Bare soil	2.7	1.68	1.68	0.88	0.57	3.38	0.54	1.08	0.58	7.46	3.56	11.61
Crop	0	40.28	58.72	3.19	17.23	46.78	9.91	0	0	0	0	0
Forest	53.33	37.32	31.43	60.82	42.74	21.52	9.33	56.72	15.79	0	0	0
Grassland	43.91	17.04	6.55	25.85	16.51	5.37	5.97	1.66	0	0	0	0
Private garden	0	3.18	0.3	3.65	6.92	13.08	39.5	5.28	31.1	3.41	4.69	3.9
Public garden	0	0	0	0	4.02	0.53	7.36	2.17	10.35	22.83	31.62	19.81
Water	0	0	0	0.06	4.99	0	0	0	1.6	12.52	0	0.37
Seminatural	97.24	54.35	37.97	86.67	59.25	26.89	15.3	58.38	15.79	0	0	0

### Bee sampling

We sampled bees using colored pan traps during six 24‐h sessions, one every 2 weeks from April 15 to July 15, 2011. This period (from early spring to early summer) comprises the peak of activity of bees and encompasses the flying period of the majority of species in the region. Pan traps offer several advantages, in particular, it has been shown to be the most efficient method for assessing bee species richness and it avoids collector bias (Westphal et al. 2008). Some studies have found that pan traps may undersample some groups such as large bees (*Bombus* and *Xylocopa* sp.) or species from the genus *Colletes* (e.g., Roulston et al. 2007; Westphal et al. 2008 and Rogers et al. 2014). However, because our main concern was to sample all 12 sites simultaneously using the same sampling effort to obtain a standardized estimate of bee species richness, we decided that pan trapping was an appropriate method for our study. Pan traps (radius = 7.25 cm, depth = 5 cm) were painted with blue, white, and yellow UV‐reflecting paints, as in Westphal et al. (2008). A set of three pan traps (one of each color) was mounted on wooden poles (1 m high) and placed at each experimental site. For each 24‐h sampling session, pan traps were filled with 400 mL of water and three drops of detergent (surfactant). Sampling was conducted under diurnal weather conditions suitable for bee activity (minimum of 15°C, low wind, no rain, and dry vegetation) to minimize variation due to climatic conditions. Insects were stored in 70% ethanol before being rinsed, dried, and mounted. Specimens were identified to the species level by experts, except for some specimens that could only be determined to the level of species group (*Bombus terrestris/lucorum*;* Halictus simplex/compressus*). In the study area, the *Bombus terrestris/lucorum* complex includes *B. terrestris* and *B. lucorum*, which are the most common species, and may also include some more rare species such as *B. cryptarum* and *B. magnus*. Six *Apis mellifera* specimens were caught but excluded from the data analysis regardless of the provenance of the individuals (hives or feral populations). Hereafter, the term “bees” thus refers to wild bee species. Taxonomy followed the nomenclature of Kuhlmann et al. (2014).

### Floral sampling

During the flowering period, five 10 m² (2 × 5 m^2^) plots were sampled within each of the 12 experimental sites. The first plot was adjacent to the three pan traps. The four additional plots were established at 50, 100, 150, and 200 m from the pan traps in a direction that was randomly chosen (north, east, south, or west). Each plot was divided into 10 cells of 1 m², and the presence/absence of each plant species was noted for each cell. Thus, by pooling the five plots at each site, the abundance of each flowering plant species was estimated with an index ranging from one to fifty. All entomophilous flowering plants were identified to the species level. We used TAXREF, the French Taxonomic Reference for the flora and fauna of metropolitan France and overseas (http://inpn.mnhn.fr/telechargement/referentielEspece/referentielTaxo). This study was not designed to study the effect of exotic plants on bee assemblage. Indeed, the maximum in the species richness of exotic plants was reached in urban sites but was only of six species. We therefore did not study the effect of exotic plants on bee assemblages.

### Data analysis

We first checked for potential spatial autocorrelation in our dataset. First, we calculated the Bray–Curtis similarity index (Magurran 2004) for wild bee assemblages and then determined the geographical distance between all pairs of sites. We performed a Mantel test with the resulting similarity and geographical distance matrices. No significant spatial autocorrelation among the sites was detected (*P *>* *0.05).

For each site, we considered seven descriptors of bee assemblages regarding taxonomic, rarity, and functional aspects: (1) bee abundance, (2) bee species richness, (3) the number of uncommon species, (4) the number of “unique species,” (5) the abundance of ground‐nesting bees, (6) the number of ground‐nesting bee species, and (7) the ratio between the numbers of aboveground and ground‐nesting species.

Information on nesting behavior was retrieved from Fortel et al. (2014) and from M. Kuhlmann (pers. comm.).

For some invertebrate groups, the existence of atlases and occurrence databases enables a rarity weight to be attributed to species at a given spatial scale (e.g., Leroy et al. 2013; for spiders). However, in most cases, such *a priori* assessment is not available, and authors evaluate the rarity status of species based on their abundance and occurrence in their own dataset (e.g., Kleijn et al. 2006; Morandin and Kremen 2013). Here, we used two measures of rarity: i) an abundance‐based indicator: the number of species that made up <1% of the total abundance in our dataset (hereafter referred to as “uncommon species”) and ii) an occurrence‐based indicator: the number of species sampled at only one site (hereafter referred to “unique species” following the terminology of Colwell and Coddington 1994). Regarding ecological traits, we focused on nesting behavior (more precisely, nest location) because this trait has been shown to determine the response of bees to urbanization (Banaszak‐Cibicka and Żmihorski 2012).

We performed generalized linear models (GLMs) to relate these seven descriptors with (1) the proportion in each land cover category; (2) plant species richness; and (3) plant abundance using R 2.14.0 software (R Development Core Team, 2012, Vienna, Austria). All GLMs were fit with a Poisson distribution and log link except for the ratio between aboveground and ground‐nesting bee species richness, which was fit with a binomial distribution and a logit link. Significance was analyzed with chi‐squared tests. The GLMs were corrected for overdispersion when it occurred. In these cases, the GLMs were refit using quasi‐Poisson errors and the *F* test (Sileshi 2006; Crawley 2007). The best‐fit models were selected by removing correlated land cover categories and by stepwise selection based on the Akaike information criterion (AIC) or the quasi‐AIC (Q‐AICc) in the case of overdispersed data (Richards 2007). The results from models with the lowest AIC are highlighted in the text, and the other results are provided in Table 2.

**Table 2:** Summary of statistical results

	Explanatory variable	*P*‐value	Significance	Overdispersion	AIC	QAICc	*F*	χ^2^	df	Estimate
Bee abundance Model: GLM with Poisson family	Crops	NS	NS							
	Forests	NS	NS							
	Grassland	NS	NS							
	Impervious surfaces	0.036	*	Yes	NA	19.10	5.82	NA	1	−0.021
	Private gardens	NS	NS							
	Plant richness	NS	NS							
	Plant abundance	NS	NS							
	Seminatural	NS	NS							
Bee species richness Model: GLM with Poisson family	Crops	0.0086	**	No	81.63	NA	NA	6.69	1	0.0081
	Forests	0.0016	**	No	78.60	NA	NA	9.93	1	0.0096
	Grassland	0.031	*	No	83.88	NA	NA	4.65	1	0.010
	Impervious surfaces	4.5 × 10^−5^	***	No	71.90	NA	NA	16.63	1	−0.012
	Private gardens	NS	NS							
	Plant richness	NS	NS							
	Plant abundance	NS	NS							
	Seminatural	0.0026	**	No	79.53	NA	NA	9.00	1	0.006
Ground‐nesting bee abundance Model: GLM with Poisson family	Crops	NS	NS							
	Forests	NS	NS							
	Grassland	NS	NS							
	Impervious surfaces	0.015	*	Yes	NA	19.00	8.43	NA	1	−0.025
	Private gardens	NS	NS							
	Plant richness	NS	NS							
	Plant abundance	NS	NS							
	Seminatural	NS	NS							
Ground‐nesting bee species richness Model: GLM with Poisson family	Crops	0.0025	**	No	82.59	NA	NA	9.11	1	0.0095
	Forests	2.5 × 10^−4^	***	No	78.30	NA	NA	13.40	1	0.011
	Grassland	0.014	*	No	85.78	NA	NA	5.92	1	0.012
	Impervious surfaces	2.4 × 10^−6^	***	No	69.48	NA	NA	22.22	1	−0.014
	Private gardens	NS	NS							
	Plant richness	NS	NS							
	Plant abundance	NS	NS							
	Seminatural	5.5 × 10^−4^	***	No	79.77	NA	NA	11.93	1	0.0072
Uncommon bee species richness Model: GLM with Poisson family	Crops	NS	NS							
	Forests	NS	NS							
	Grassland	NS	NS							
	Impervious surfaces	0.002	**	No	64.37	NA	NA	9.34	1	−0.013
	Private gardens	NS	NS							
	Plant richness	NS	NS							
	Plant abundance	NS	NS							
	Seminatural	0.035	*	No	69.29	NA	NA	4.41		0.0064
Ratio above/belowground Model: GLM with Binomial family	Crops	0.022	*	No	34.38	NA	NA	5.22	1	−0.043
	Forests	0.03	*	No	31.03	NA	NA	8.57	1	−0.045
	Grassland	NS	NS							
	Impervious surfaces	0.0008	***	No	28.39	NA	NA	11.22	1	0.043
	Private gardens	NS	NS							
	Plant richness	NS	NS							
	Plant abundance	NS	NS							
	Seminatural	0.0052	**	No	31.81	NA	NA	7.79	1	−0.031
Unique bee species richness Model: GLM with Poisson family	Crops	NS	NS							
	Forests	0.025	*	No	45.31	NA	NA	4.99	1	0.017
	Grassland	0.016	*	No	44.52	NA	NA	5.45	1	0.027
	Impervious surfaces	NS	NS							
	Private gardens	NS	NS							
	Plant richness	NS	NS							
	Plant abundance	NS	NS							
	Seminatural	0.011	*	No	43.98	NA	NA	6.25	1	0.012

Fisher's test and Q‐AICc were used when overdispersion was present.

Significant effects are indicated by *P <* *0.05, **P ≤* *0.01, ***P ≤* *0.005.

To conduct co‐inertia analyses, we first removed plant and bee species that were present at only a single site to downweight the effect of uncommon species. Using the ade4 package (Dray and Dufour 2007), we performed a correspondence analysis (CA) on wild bee abundance per site (12 sites × 55 species) and principal component analyses (PCAs) on the flowering plant assemblage composition (12 sites × 91 plant species) and landscape land cover composition within a 500 m radius (12 sites × 8 landscape variables). We then performed co‐inertia analyses between the wild bee assemblage structure and both (1) the flowering plant assemblage structure and (2) the landscape land cover composition within a 500 m radius. Co‐inertia analysis is basically a method to couple two data tables (Dray et al. 2003). This method projects the two tables in the same factorial plane that maximizes the covariance between these tables. For example, in a first table, experimental sites are characterized by their fauna assemblages, and in a second table, experimental sites are characterized by environmental variables. Two multivariate analyses are performed on these tables (for example, a CA and a PCA). The co‐inertia analysis projects simultaneously on the same co‐inertia space the two independent previous analyses (CA and PCA) to maximise their covariance. The significance of a co‐inertia (the strength of the covariance) can be thus obtained using a Monte Carlo random permutation test (999 permutations). This method is a powerful tool to study the link between species and their environment (Dray et al. 2003; Thioulouse et al. 2004) and is particularly efficient to simultaneously study environmental and fauna descriptors (Dolédec and Chessel 1994; Dray et al. 2003).

## Results

### Wild bee fauna

A total of 1104 individuals representing 74 species from six bee families (Andrenidae, Apidae, Colletidae, Halictidae, Megachillidae, and Melittidae) were collected (Table 3; Table S1). The most abundant species was *Lasioglossum malachurum* (221 individuals), representing 20.02% of the total abundance. In contrast, 28 species were represented by a single individual. Species richness ranged from seven (site 9) to 26 species (sites 5 and 6). Bee abundance ranged from 11 (site 9) to 287 individuals (site 5). Thirty‐five species were “unique,” that is, present at only one site. The number of unique species ranged from one (sites 9 and 11) to eight (site 1). Fifty‐six species were uncommon, *that is,* representing <1% of the total abundance in our dataset. The number of uncommon species ranged from two (sites 9 and 11) to 13 species (site 1). There were nine aboveground‐nesting species, representing 4.1% of the total abundance. The ratio between the numbers of aboveground and ground‐nesting species ranged from 0% (site 2, 4, and 9) to 40.0% (site 10).

**Table 3:** Description of bee communities (abundance and species richness). Uncommon species are species representing <1% of the total abundance in our dataset. Unique species are species sampled at only one site

	Site1	Site2	Site3	Site4	Site5	Site6	Site7	Site8	Site9	Site10	Site11	Site12
Bee abundance	73	116	145	71	287	122	54	76	11	89	35	25
Bee species richness	22	21	22	19	26	26	21	23	7	14	10	10
Ground‐nesting bee abundance	71	116	144	71	284	120	53	75	11	54	34	20
Ground‐nesting bee richness	21	21	21	19	24	25	20	22	7	10	9	8
Uncommon species	13	11	9	5	11	12	12	10	2	6	2	6
Unique bee species	8	2	2	2	4	3	3	5	1	2	1	2
Ratio above/belowground	0.047	0.000	0.047	0.000	0.083	0.040	0.050	0.045	0.000	0.400	0.110	0.250

### Influence of the urbanization gradient

**Figure 2:**
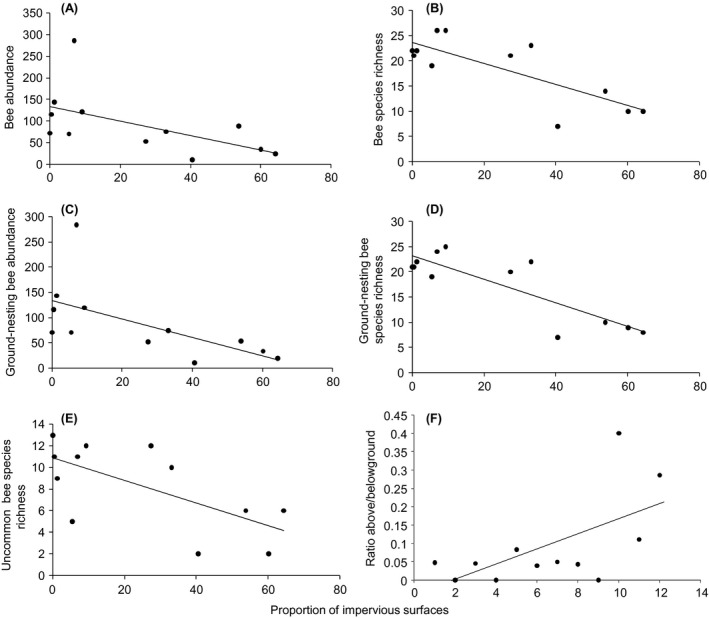
Linear relationship between (*x*‐axis) the proportion of impervious surfaces and (*y*‐axis) (A) bee abundance, (B) bee species richness, (C) ground‐nesting bee abundance, (D) ground‐nesting bee species richness, (E) Uncommon bee species, and (F) ratio between aboveground and belowground bee species richness.

**Figure 3:**
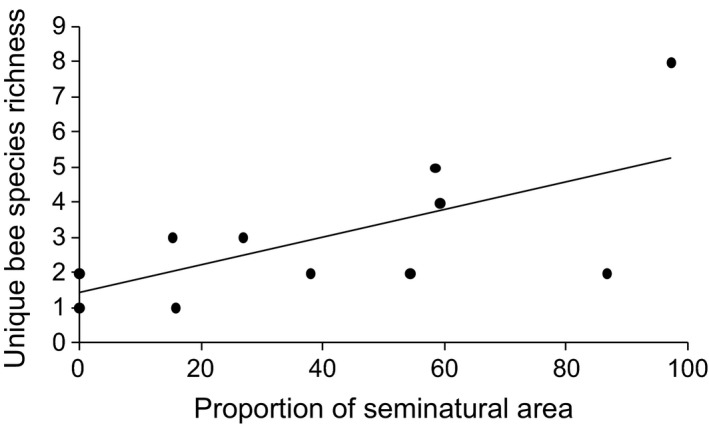
Linear relationship between (*x*‐axis) the proportion of seminatural areas and (*y*‐axis) the species richness of unique bees.

An increase in the proportion of impervious surfaces at the landscape scale led to a significant decrease in wild bee abundance and species richness, uncommon species richness, and ground‐nesting bee abundance and species richness (Table 2, Fig. 2). Conversely, we observed a significant increase in aboveground bee species richness as the proportion of impervious surfaces increased (Table 2, Fig. 2). Finally, the species richness of unique species significantly increased as the proportion of seminatural habitats at the landscape scale increased (Table 2, Fig. 3).

### Effect of the landscape composition on wild bee assemblages

**Figure 4:**
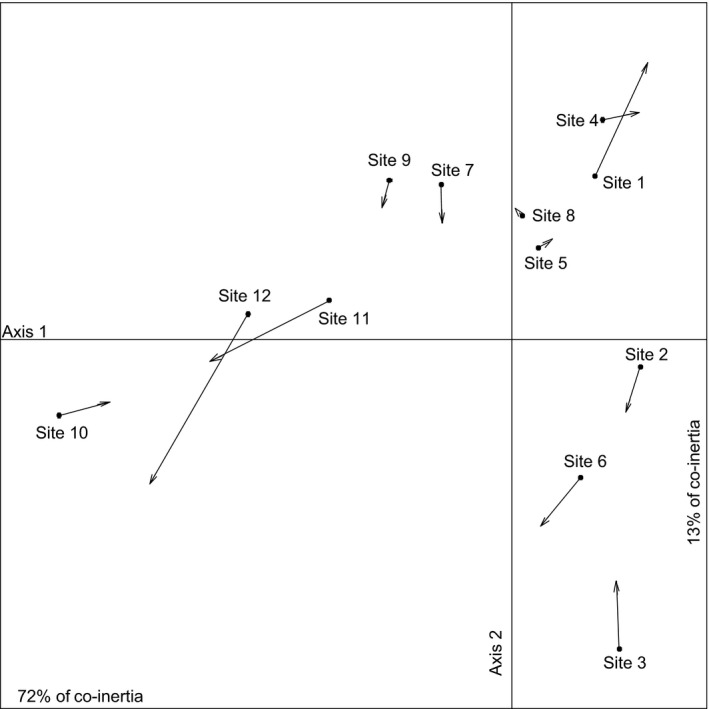
Projections of study sites on the first factorial plane of the co‐inertia analysis. Circles represent the projection from the correspondence analysis computed with bee data. Arrows represent the projection from the component analysis computed with landscape composition data. The numbers represent the different sites. A shorter arrow indicates a strong covariance between fauna and landscape descriptors within an experimental site. The inertia explained by the two first factorial axes is provided. Axis 1 that opposes densely urbanized sites to agricultural and seminatural‐dominated sites explains most of the inertia (72%). Axis 2 explaining 13% of the co‐inertia opposes agricultural sites to seminatural ones dominated by grasslands and forests.

**Figure 5:**
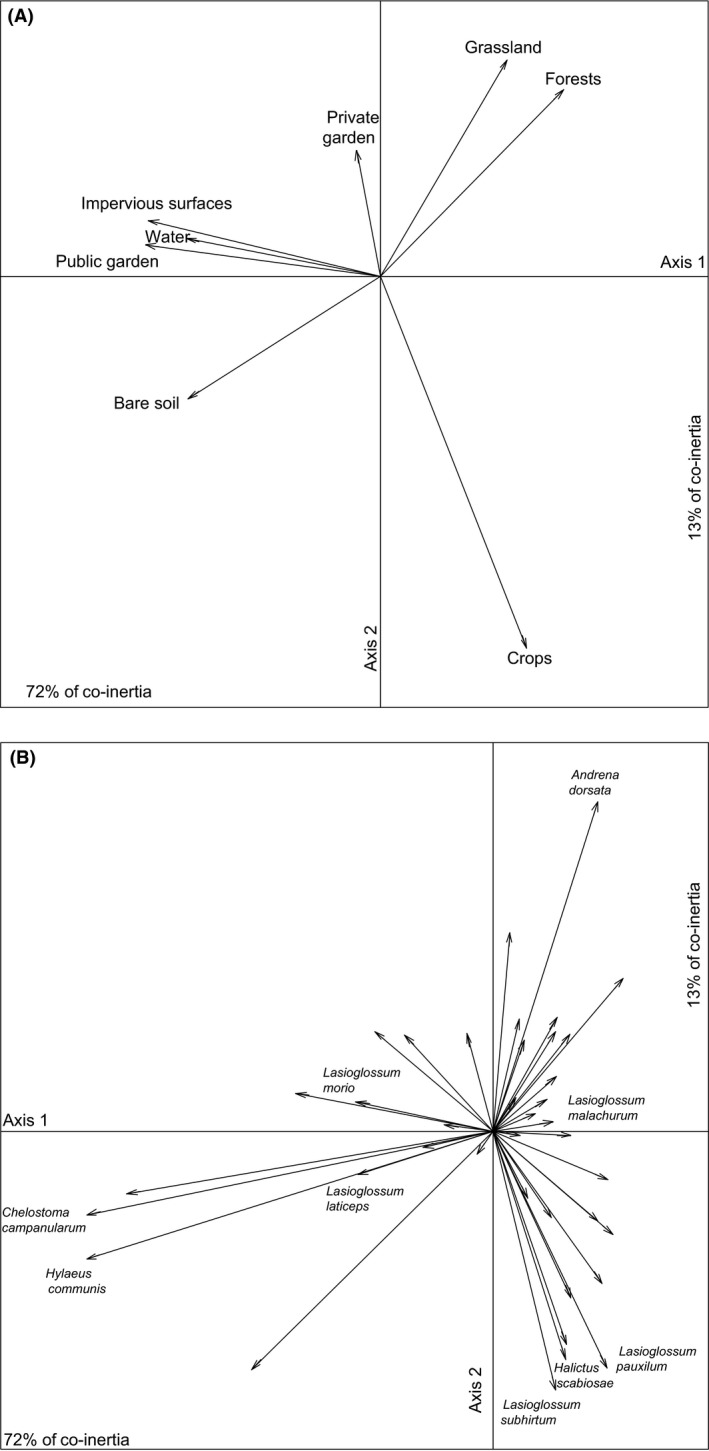
(A) Projection of landscape composition on the first factorial plane of the co‐inertia analysis. Axis 1 explaining 72% of the co‐inertia opposes densely urbanized sites dominated by impervious surfaces to seminatural sites dominated by grassland and forest and agricultural site dominated by crops. Axis 2 explaining 13% of the co‐inertia opposes agricultural sites to seminatural ones dominated by grassland and forest. (B) Projections of insect species on the first factorial plane of the co‐inertia analysis. The species that explained the most inertia are indicated. The inertia explained by the two first factorial axes is provided. Axis 1 explaining 72% of the co‐inertia opposes densely urbanized sites characterized by the presence of *Chelostoma campanularum* and *Hylaeus communis* to seminatural sites characterized by the presence of *Andrena dorsata* and to the agricultural sites characterized by the presence of *Lasioglossum pauxilum, L. subhirtum,* and *Halictus scabiosae*. Axis 2 explaining 13% of the co‐inertia opposes seminatural sites characterized by the presence of *Andrena dorsata* and agricultural sites characterized by the presence of *Lasioglossum pauxilum, L. subhirtum,* and *Halictus scabiosae*.

The co‐inertia analysis (Fig. 4) matched the simultaneous positions of the 12 sites derived from covariance of the PCA on landscape composition to the positions derived from covariance of the CA on bee assemblages. The result of the Monte Carlo permutation test was highly significant (*P *=* *0.001). The first axis of the co‐inertia plane accounted for 72% of the total inertia, whereas the second axis accounted for 13%. The first axis clearly opposed sites dominated by impervious surfaces to those dominated by crops, forests, and grasslands and can thus be interpreted as an urbanization gradient (Fig. 5A). When considering species contributions to the first axis, *Chelostoma campanularum, Lasioglossum morio*,* Hylaeus communis*,* Lasioglossum laticeps, and Lasioglossum malachurum* were species that contributed the most (Fig. 5B). Among those species, *Chelostoma campanularum* and *Hylaeus communis* were particularly associated with urbanized sites. The second axis of the co‐inertia plane opposed crop‐dominated sites to forested and grassland‐dominated sites. *Lasioglossum pauxillum, Lasioglossum subhirtum, Halictus scabiosae*, and *Andrena dorsata* strongly contributed to the second axis (Fig. 5B). Among those, *Halictus scabiosae*,* Lasioglossum pauxillum,* and *Lasioglossum subhirtum* were associated with crop‐dominated sites, whereas *Andrena dorsata* and *Lasioglossum calceatum* were associated with forested and grassland‐dominated sites.

### Impact of the local flowering plants on bee assemblages

In all 12 experimental sites, we identified 195 entomophilous flowering plant species representing 53 families (Table S2). The most common species was *Taraxacum ruderale*, with 74 occurrences over all of the study sites. In comparison, 106 species were only present at a single site. The total number of species ranged from 9 (site 12) to 69 (site 10). We did not detect any effect of plant species richness or plant abundance on the different descriptors of bee assemblages (Table 2). The co‐inertia analysis between the composition of local flowering plant communities and wild bee assemblages was also not significant (Monte Carlo permutation test *P *>* *0.05).

## Discussion

Our results indicate that increasing urbanization as measured by an increasing proportion of impervious surfaces within a 500 m radius resulted in an important decrease in wild bee abundance and species richness. Ground‐nesting bees and uncommon species were more impacted by urbanization than aboveground‐nesting species. Furthermore, our results show that the presence of permanent grassland areas promotes the maintenance of uncommon species. Finally, we did not find any relation between plant species richness, plant abundance or local‐scale plant assemblage composition and the descriptors of bee assemblages studied.

### Wild bee assemblages

This study provides some initial insights into the species composition of bee communities in the Île‐de‐France region, for which knowledge of bee fauna is scarce (but see Deguines et al. 2012 and Shwartz et al. 2013 for studies at the levels of higher taxa and morphospecies; and Geslin et al. 2016). We collected 74 bee species in our 12 sites between mid‐April and mid‐July 2011, which represents a small proportion (8%) of the 926 species that have been recorded in France (Kuhlmann et al. 2014). This is largely because wild bee diversity is generally higher in Mediterranean regions throughout the world (Michener 2007), such as in southern France. Moreover, a multiyear study or the simultaneous use of complementary sampling methods (netting or trap nests) would also have likely led to a greater number of detected species (Westphal et al. 2008). Indeed, Grundel et al. (2011) and Banaszak et al. (2014) have stressed the importance of exhaustive sampling in assessing the total number of bee species at a study site, primarily due to high spatiotemporal variability in bee assemblages (e.g., Rollin et al. 2015).

Regarding taxonomical aspects, the dominance of nonparasitic halictids (*Lasioglossum* and *Halictus* spp.) appears to be a common feature in bee assemblages (e.g., Marini et al. 2012; Morandin and Kremen 2013; Fortel et al. 2014; Geroff et al. 2014; Saunders and Luck 2014; Torné‐Noguera et al. 2014; Pisanty and Mandelik 2015). These species are especially well caught by pan traps, but high abundance of these species is also observed when bees are sampled by netting (e.g., Rollin et al. 2015). More specifically, a high abundance of the social species *Lasioglossum malachurum* has been also observed in other contexts, such as in agricultural areas in Israel (Pisanty and Mandelik 2015), and France (Rollin et al. 2015).

### Impact of urbanization and impervious surfaces

We found an important decrease in bee abundance and species richness along the urbanization gradient, which was measured by an increase in the proportion of impervious surfaces within a 500 m radius. Along the urbanization gradient, the increase in the proportion of impervious surfaces was directly correlated with the decline in the proportion of forested area, crops, or grasslands. Such multicolinearity is common in studies of urbanization gradients (see Bates et al. 2011), but our results clearly show that the proportion of impervious surfaces was the primary explanatory variable and was linked with wild bee assemblages.

This result is consistent with the findings of several previous studies performed along urbanization gradients. For example, Ahrné et al. (2009) showed a decrease in bumblebee richness (but not abundance) in the urban area of Stockholm; Bates et al. (2011) showed that urban sites support a smaller richness and abundance of pollinators than rural ones; Matteson et al. (2008) showed a reduced bee richness in urban gardens of New York compared to New York state and New Jersey; and Zanette et al. (2005) showed a decrease in the abundance of wild bees with increasing urbanization (see Hernandez et al. 2009 for a review). Urbanization is one of the main drivers of the destruction of natural environments, resulting in habitat loss for pollinators (Goddard et al. 2010). Specifically, impervious surfaces reduce the availability of resources and nesting sites and impede ground‐nesting species from reproducing in cities (McIntyre and Hostelter, 2001). Moreover, urbanization often leads to degradation of the few available nesting sites through soil drying or compaction (Cane et al. 2006). Nesting requirements have been shown to be a good predictor of the response of bee species to habitat alteration, with ground‐nesting species being especially sensitive to urbanization because they require bare soil surfaces to establish their nests (Cane et al. 2006; Xie et al. 2013). For example, Cane et al. (2006) showed that cavity nesters were overrepresented in an urban matrix, likely due to the numerous nesting opportunities for those species (*e.g.,* holes in building walls). This consideration might explain the decrease of ground‐nesting species, such as *Lasioglossum* and *Halictus* spp. observed in densely urbanized sites. This might also explain why some aboveground‐nesting species, such as *Hylaeus communis* and *Megachile willughbiella*, are present in urban matrices. On the other hand, some wildlife‐friendly practices observed close to our urban sites, such as “hotels” for bees built with bundles of cardboard tubes or reeds (Mader et al. 2010), might have locally promoted the presence of these species over ground‐nesting species (Fortel et al. 2016).

Body size and flight abilities are often correlated among bees (Araújo et al. 2004; Stang et al. 2006). Populations of small‐bodied pollinators with limited flight distances and foraging ranges generally tend to decrease in urban and suburban environments (Banaszak‐Cibicka and Żmihorski 2012). This was the case in our survey, where small solitary bee species such as those belonging to the Halictini tribe were proportionally less present in urbanized sites. Inversely, large‐bodied species often exhibit good flight abilities and a large foraging range (Gathmann and Tscharntke 2002; Greenleaf et al. 2007). Their ability to fly relatively long distances (up to ~1 km; Zurbuchen et al. 2010) makes them less vulnerable to the habitat fragmentation induced by increasing urbanization. At the scale at which the study was conducted, large‐bodied species seemed thus to be able to fly between rewarding patches and nesting sites (Matteson and Langellotto 2009). However, because dispersion range is larger than the landscape scale we considered (500 m), we cannot exclude a potential effect of the proportion of impervious surfaces on large‐bodied bee species at a larger landscape scale. For example, Ahrné et al. (2009) found a stronger negative correlation between bumblebee diversity (bumblebees are large‐bodied species compared to the Halictini tribe, for example) and impervious surfaces for a landscape window of 1000 m compared to those of 300 m and 500 m. As similar to Andersson et al. (2009) and Lowe et al. (2014), we believe that using multivariate gradients for future studies (with different landscape scales) will improve our knowledge of the impact of urbanization on bee assemblages.

### Importance of the heterogeneity of the landscape composition

Our results also highlight the importance of maintaining some heterogeneity in landscape composition to preserve wild bees. We found that seminatural areas promoted the maintenance of uncommon species or habitat‐specific species (defined here as unique species). Similarly, the fauna from some of our crop‐dominated sites was primarily composed of ground‐nesting and food generalist species that were virtually absent in urbanized and grassland‐dominated sites (e.g., *Halictus scabiosae* and *Lasioglossum subhirtum*). Thus, seminatural habitats or even agricultural areas should be maintained around cities to promote the conservation of bee diversity. Also, in other urban contexts, cities might be really heterogeneous with a high diversity of habitats. This could explain why other cities such as Lyon, France (see Fortel et al. 2014), harbored a higher bee richness than in our study.

### Local‐scale and landscape‐scale factors

The link between bee and plant species richness has been well documented in the scientific literature for croplands (e.g., Holzschuh et al. 2007; Kennedy et al. 2013), grasslands (e.g., Fründ et al. 2010; Ebeling et al. 2012), and urban habitats (e.g., Frankie et al. 2009; Kearns and Oliveras 2009). Thus, in cities, enhancing flowering plant species richness is more likely to positively impact bee species richness and could mitigate the negative effect of urbanization (Kearns and Oliveras 2009; Hennig and Ghazoul 2011b; Wojcik and McBride 2011). For example, cities with abundant and diverse flowering plants might support a pollinator assemblage comparable to that of surrounding natural habitats (Fetridge et al. 2008).

We therefore hypothesized that local flowering plants might significantly influence the composition of bee assemblages in our sites. However, our results did not indicate any effect of plant species richness, species abundance, or the composition of local plant assemblages on bees. In intensely managed environments, the structure of plant assemblages depends strongly on gardening practices (Politi‐Bertoncini et al. 2012). Plant assemblages vary over short periods and might be more reflective of economic and social influences rather than their ecological value to pollinators (Hope et al. 2010). Thus, the structure of urban plant assemblages may not directly relate the structure of pollinator assemblages. Indeed, urbanization promotes the loss of native species and their replacement by non‐native ones (e.g., Bergerot et al. 2010; Goddard et al. 2010); for example, city gardens are often planted with horticultural or ornamental plants, which artificially increase species richness and change the composition of plant assemblages (McKinney 2008; Perre et al. 2011). In some cases, pollinators have been described to visit those exotic plants within cites (Hanley et al. 2014; Salisbury et al. 2015), even if native plants seemed to be preferred (Corbet et al. 2001; Williams et al. 2011) and to be a better descriptor of pollinator communities (Burghardt et al. 2009; Pardee and Philpott 2014). Nevertheless, the effect of the exotic vegetation on pollinator assemblages in urban ecosystems is still in debate in the literature (Goddard et al. 2010) and even if this study was not designed to state on this issue, it stressed the need for future research linking pollinator communities and urban exotic flora.

As illustrated by Matteson et al. 2012, plant assemblages vary not only over short periods but also over short spatial scales in cities. This study notably showed that very small‐scale variations (30 m) in the vegetation cover may strongly influence flower‐visiting insects in New York. Once again, it highlights the importance of considering a wide range of spatial scales in future studies.

The sampling technique used in the current study might also have induced a bias in the results. Several studies have shown that pan trap attractiveness might vary with flower abundance in the surroundings, with pan trap effectiveness decreasing as floral resource availability increases (Wilson et al. 2008; Baum and Wallen 2011; Cane et al. 2013). In our study, pan traps placed in flower‐rich urban parks may have been less attractive compared to those placed in agricultural and seminatural sites that locally offer fewer flowers. Thus, because differences in floral resource availability might influence how effective pan traps are, captures might not totally reflect the diversity of the local bee fauna, thus leading to a lack of correlation between the composition of bee assemblages and the local flower composition. When relating bee and flower assemblages, netting appears to be the best sampling method (Westphal et al. 2008; Popic et al. 2013).

Taken together, these results indicate the difficulty of generalizing the relationships between plant and bee assemblages in urban environments. For future studies, it might be interesting to analyze the functional traits of those assemblages and the relations between plants and pollinator functional traits along urbanization gradients.

## Conclusion

Wild bee abundance, species richness, and assemblage composition were all negatively correlated with the proportion of impervious surfaces at the landscape scale, but no effects of local flower composition were underlined. Here, uncommon bees and ground‐nesting bees were particularly sensitive to increasing urbanization, whereas unique species were primarily found in seminatural‐dominated sites. This species loss might have direct implications for urban ecosystems. A loss of species often leads to a loss of interactions and thus a loss of ecological functions, both of which are key providers of ecosystem services (Fontaine et al. 2006; Tylianakis et al. 2010). Given the growing interest in urban agriculture (Matteson and Langellotto 2009), the loss of pollinating functions within cities might impair the development of crop systems in urban gardens. This concern is particularly important for cities such as Paris, where the growth of urban areas often occurs at the expense of agricultural land (Torre et al. 2013). In this context, urban agriculture might become increasingly important for food security and the provision of fresh products to inhabitants (Brown and Jameton 2000; Pawelek et al. 2009).

Our results indicate a correlation between the proportion of impervious surfaces within a 500 m radius and the structure of bee assemblages in the Île‐de‐France region. In this context, determining a precise threshold for the proportion of impervious surfaces above which permanent changes occur in bee assemblages could greatly improve conservation measures for pollinating insects and plants within cities and should have implications for future urban landscape planning (Kato and Ahern 2011). As the world continues to change rapidly and becomes increasingly urbanized, new conservation policies are needed to preserve the ability of anthropized areas to provide habitats for pollinators. In cities, it has been suggested that the proportion of impervious surfaces might be reduced through the installation of green roofs (Colla et al. 2009). These types of management practice might promote the survival of ground‐nesting bees.

Our data do not suggest an impact of local flora assemblages on bee assemblages. Neither the composition, abundance nor richness of plant assemblages affected the descriptors of bee assemblages in this study. However, as we stated, this lack of correlation might be due to the sampling method or the scale considered. We suggest here to study the link between plant assemblages and bee assemblages at a wide range of spatial scales from the very local scale (30 m) to the landscape scale (500 m). Finally, even if pollinators might visit exotic plants, the favouring of pollinator‐attractive flora within city gardens through the inclusion of native flora has been previously shown to have a positive impact on local pollinating fauna (Pawelek et al. 2009; Pardee and Philpott 2014), and such a modification in local flora could easily be implemented in cities such as Paris, as it might have a positive impact on other taxonomic groups of pollinators (e.g., butterflies, Burghardt et al. 2009).

Further studies linking pollinator diversity and urbanization are needed, particularly if we want to compare the findings of cities with emerging global trends; we strongly encourage the development of multiyear and multiscale studies.

## Conflict of Interest

None declared.

## Supporting information


**Table S1‐1,‐2.** Abundance of bee species in each site and the total number of sites where each species has been caught.

**Table 2‐1,‐2,‐3,‐4.** Species distribution of flowering plant species in each experimental site.
