# Supplementary material for: The proportion of impervious surfaces at the landscape scale structures wild bee assemblages in a densely populated region
Source: Ecol Evol. 2016 Aug 25;6(18):6599–615. doi: 10.1002/ece3.2374 (PMC5058531; doi:10.1002/ece3.2374)
Supplement: Supplementary file 1 — Table S1‐1,‐2. Abundance of bee species in each site and the total number of sites where each species has been caught. [file ECE3-6-6599-s001.docx]

**Table S1-1**: Abundance of bee species in each site and the total number of sites where each species has been caught. Above-ground nesting bees are highlighted in grey.

| **Family** | **Genus** | **Species** | **site1** | **site2** | **site3** | **site4** | **site5** | **site6** | **site7** | **site8** | **site9** | **site10** | **site11** | **site12** |
| --- | --- | --- | --- | --- | --- | --- | --- | --- | --- | --- | --- | --- | --- | --- |
|  |  |  |  |  |  |  |  |  |  |  |  |  |  |  |
| Andrenidae | *Andrena* | *angustior* | 0 | 2 | 0 | 0 | 0 | 0 | 4 | 0 | 1 | 0 | 0 | 0 |
| Andrenidae | *Andrena* | *bicolor* | 0 | 1 | 1 | 0 | 5 | 1 | 1 | 0 | 0 | 0 | 0 | 0 |
| Andrenidae | *Andrena* | *bimaculata* | 0 | 1 | 0 | 0 | 0 | 0 | 0 | 0 | 0 | 0 | 0 | 0 |
| Andrenidae | *Andrena* | *cineraria* | 0 | 0 | 0 | 0 | 0 | 0 | 0 | 0 | 0 | 0 | 1 | 0 |
| Andrenidae | *Andrena* | *dorsata* | 1 | 0 | 0 | 5 | 0 | 0 | 1 | 0 | 0 | 0 | 0 | 0 |
| Andrenidae | *Andrena* | *flavipes* | 1 | 8 | 0 | 6 | 10 | 5 | 0 | 1 | 0 | 1 | 0 | 0 |
| Andrenidae | *Andrena* | *fulva* | 0 | 0 | 0 | 0 | 0 | 0 | 1 | 0 | 0 | 0 | 0 | 0 |
| Andrenidae | *Andrena* | *gravida* | 0 | 1 | 0 | 0 | 0 | 0 | 1 | 0 | 0 | 0 | 0 | 0 |
| Andrenidae | *Andrena* | *minutula* | 0 | 0 | 0 | 0 | 6 | 2 | 1 | 0 | 0 | 0 | 0 | 2 |
| Andrenidae | *Andrena* | *minutuloides* | 0 | 0 | 0 | 0 | 0 | 0 | 0 | 0 | 0 | 0 | 0 | 1 |
| Andrenidae | *Andrena* | *nigroaenea* | 0 | 0 | 0 | 0 | 0 | 2 | 1 | 2 | 0 | 1 | 0 | 0 |
| Andrenidae | *Andrena* | *nitida* | 0 | 0 | 1 | 0 | 0 | 0 | 0 | 1 | 0 | 0 | 0 | 0 |
| Andrenidae | *Andrena* | *pandellei* | 1 | 0 | 2 | 0 | 0 | 0 | 0 | 0 | 0 | 0 | 0 | 0 |
| Andrenidae | *Andrena* | *praecox* | 0 | 0 | 1 | 0 | 0 | 0 | 0 | 0 | 0 | 0 | 0 | 0 |
| Andrenidae | *Andrena* | *semilaevis* | 0 | 0 | 0 | 0 | 0 | 0 | 0 | 0 | 0 | 0 | 0 | 2 |
| Andrenidae | *Andrena* | *similis* | 0 | 0 | 0 | 0 | 0 | 0 | 0 | 1 | 0 | 0 | 0 | 0 |
| Andrenidae | *Andrena* | *subopaca* | 0 | 1 | 0 | 0 | 0 | 0 | 0 | 0 | 0 | 0 | 0 | 0 |
| Andrenidae | *Panurgus* | *calcaratus* | 1 | 0 | 0 | 0 | 0 | 0 | 0 | 0 | 0 | 0 | 0 | 0 |
| Andrenidae | *Panurgus* | *dentipes* | 0 | 0 | 0 | 0 | 1 | 1 | 0 | 0 | 0 | 0 | 0 | 0 |
| Apidae | *Bombus* | *hortorum* | 0 | 1 | 1 | 0 | 0 | 0 | 0 | 0 | 0 | 0 | 0 | 0 |
| Apidae | *Bombus* | *lapidarius* | 5 | 2 | 4 | 2 | 0 | 7 | 0 | 4 | 0 | 0 | 0 | 0 |
| Apidae | *Bombus* | *pascuorum* | 0 | 1 | 0 | 0 | 2 | 0 | 1 | 0 | 0 | 0 | 0 | 0 |
| Apidae | *Bombus* | *sylvestris* | 0 | 0 | 0 | 0 | 0 | 0 | 1 | 0 | 0 | 0 | 0 | 0 |
| Apidae | *Bombus* | *terrestris & lucorum* | 2 | 0 | 3 | 0 | 2 | 10 | 3 | 11 | 0 | 0 | 0 | 0 |
| Apidae | *Nomada* | *bifasciata* | 0 | 0 | 0 | 0 | 1 | 0 | 0 | 0 | 0 | 0 | 0 | 0 |
| Apidae | *Nomada* | *flavugottata* | 0 | 0 | 0 | 0 | 0 | 1 | 0 | 0 | 0 | 0 | 0 | 2 |
| Apidae | *Nomada* | *signata* | 1 | 0 | 0 | 0 | 0 | 0 | 0 | 0 | 0 | 0 | 0 | 0 |
| Apidae | *Tetralonia* | *malvae* | 4 | 0 | 0 | 0 | 0 | 0 | 0 | 0 | 0 | 0 | 0 | 0 |
| Apidae | *Xylocopa* | *violacea* | 0 | 0 | 0 | 0 | 0 | 0 | 0 | 1 | 0 | 0 | 0 | 0 |
| Colletidae | *Colletes* | *cunicularius* | 1 | 0 | 0 | 0 | 0 | 0 | 0 | 0 | 0 | 0 | 0 | 0 |
| Colletidae | *Hylaeus* | *communis* | 0 | 0 | 0 | 0 | 0 | 0 | 0 | 0 | 0 | 5 | 0 | 2 |
| Halictidae | *Halictus* | *confusus* | 0 | 0 | 0 | 0 | 0 | 0 | 0 | 1 | 0 | 0 | 0 | 0 |
| Halictidae | *Halictus* | *maculatus* | 0 | 11 | 1 | 2 | 1 | 0 | 0 | 0 | 0 | 0 | 0 | 0 |
| Halictidae | *Halictus* | *rubicundus* | 2 | 3 | 0 | 1 | 1 | 1 | 2 | 1 | 0 | 0 | 0 | 0 |
| Halictidae | *Halictus* | *scabiosae* | 0 | 3 | 13 | 0 | 2 | 4 | 2 | 1 | 0 | 0 | 0 | 0 |
| Halictidae | *Halictus* | *sexcinctus* | 1 | 0 | 0 | 0 | 0 | 0 | 0 | 0 | 0 | 0 | 0 | 0 |
| Halictidae | *Halictus* | *simplex* | 0 | 0 | 14 | 0 | 8 | 1 | 0 | 1 | 0 | 0 | 0 | 0 |
| Halictidae | *Halictus* | *tumulorum* | 2 | 6 | 0 | 15 | 7 | 9 | 2 | 2 | 0 | 0 | 2 | 0 |

**Tables S1-2**: Abundance of bee species in each site and the total number of sites where each species has been caught. Above-ground nesting bees are highlighted in grey.

| **Family** | **Genus** | **Species** | **site1** | **site2** | **site3** | **site4** | **site5** | **site6** | **site7** | **site8** | **site9** | **site10** | **site11** | **site12** |
| --- | --- | --- | --- | --- | --- | --- | --- | --- | --- | --- | --- | --- | --- | --- |
|  |  |  |  |  |  |  |  |  |  |  |  |  |  |  |
| Halictidae | *Lasioglossum* | *aeratum* | 4 | 0 | 0 | 0 | 0 | 0 | 0 | 0 | 0 | 0 | 0 | 0 |
| Halictidae | *Lasioglossum* | *albipes* | 5 | 0 | 0 | 0 | 0 | 0 | 0 | 0 | 0 | 0 | 0 | 0 |
| Halictidae | *Lasioglossum* | *bluethgeni* | 0 | 0 | 0 | 0 | 0 | 0 | 0 | 0 | 1 | 0 | 0 | 0 |
| Halictidae | *Lasioglossum* | *calceatum* | 23 | 9 | 17 | 15 | 6 | 9 | 5 | 14 | 2 | 0 | 1 | 0 |
| Halictidae | *Lasioglossum* | *fulvicorne* | 0 | 1 | 2 | 1 | 2 | 1 | 0 | 3 | 0 | 0 | 0 | 0 |
| Halictidae | *Lasioglossum* | *glabriusculum* | 0 | 8 | 1 | 0 | 0 | 1 | 0 | 0 | 0 | 0 | 0 | 0 |
| Halictidae | *Lasioglossum* | *interruptum* | 0 | 0 | 0 | 0 | 0 | 1 | 0 | 0 | 0 | 0 | 0 | 0 |
| Halictidae | *Lasioglossum* | *laticeps* | 0 | 0 | 9 | 1 | 13 | 3 | 2 | 6 | 1 | 18 | 2 | 3 |
| Halictidae | *Lasioglossum* | *lativentre* | 0 | 0 | 0 | 1 | 0 | 0 | 0 | 0 | 0 | 0 | 0 | 0 |
| Halictidae | *Lasioglossum* | *leucozonium* | 2 | 0 | 7 | 1 | 2 | 0 | 0 | 0 | 0 | 0 | 0 | 0 |
| Halictidae | *Lasioglossum* | *lineare* | 0 | 0 | 2 | 0 | 0 | 0 | 0 | 0 | 0 | 0 | 0 | 0 |
| Halictidae | *Lasioglossum* | *majus* | 1 | 1 | 0 | 0 | 0 | 0 | 0 | 0 | 0 | 0 | 0 | 0 |
| Halictidae | *Lasioglossum* | *malachurum* | 0 | 40 | 14 | 5 | 137 | 20 | 0 | 2 | 0 | 1 | 1 | 1 |
| Halictidae | *Lasioglossum* | *mediterraneum* | 0 | 0 | 0 | 0 | 0 | 0 | 0 | 1 | 0 | 0 | 0 | 0 |
| Halictidae | *Lasioglossum* | *morio* | 7 | 2 | 3 | 1 | 22 | 7 | 4 | 6 | 4 | 20 | 20 | 2 |
| Halictidae | *Lasioglossum* | *nitidulum* | 0 | 0 | 0 | 8 | 27 | 1 | 16 | 4 | 1 | 8 | 3 | 9 |
| Halictidae | *Lasioglossum* | *pallens* | 0 | 0 | 0 | 0 | 0 | 0 | 1 | 0 | 0 | 0 | 0 | 0 |
| Halictidae | *Lasioglossum* | *pauperatum* | 0 | 0 | 0 | 0 | 0 | 2 | 0 | 0 | 0 | 0 | 0 | 0 |
| Halictidae | *Lasioglossum* | *pauxillum* | 0 | 12 | 34 | 2 | 4 | 3 | 0 | 0 | 0 | 1 | 3 | 0 |
| Halictidae | *Lasioglossum* | *politum* | 0 | 2 | 1 | 2 | 19 | 2 | 1 | 7 | 1 | 0 | 1 | 0 |
| Halictidae | *Lasioglossum* | *punctatissimum* | 4 | 0 | 0 | 0 | 0 | 0 | 0 | 0 | 0 | 0 | 0 | 0 |
| Halictidae | *Lasioglossum* | *puncticolle* | 0 | 0 | 0 | 0 | 1 | 0 | 0 | 0 | 0 | 0 | 0 | 0 |
| Halictidae | *Lasioglossum* | *pygmaeum* | 2 | 0 | 0 | 1 | 5 | 0 | 0 | 4 | 0 | 1 | 0 | 0 |
| Halictidae | *Lasioglossum* | *sabulosum* | 0 | 0 | 0 | 0 | 0 | 0 | 0 | 0 | 0 | 1 | 0 | 0 |
| Halictidae | *Lasioglossum* | *sexstrigatum* | 0 | 0 | 0 | 0 | 0 | 0 | 0 | 1 | 0 | 0 | 0 | 0 |
| Halictidae | *Lasioglossum* | *subhirtum* | 2 | 0 | 13 | 1 | 0 | 26 | 0 | 0 | 0 | 0 | 0 | 0 |
| Halictidae | *Lasioglossum* | *villosulum* | 0 | 0 | 0 | 0 | 1 | 0 | 0 | 1 | 0 | 2 | 0 | 0 |
| Halictidae | *Lasioglossum* | *zonulum* | 0 | 0 | 0 | 1 | 0 | 0 | 0 | 0 | 0 | 0 | 0 | 0 |
| Megachilidae | *Chelostoma* | *campanularum* | 0 | 0 | 0 | 0 | 0 | 0 | 1 | 0 | 0 | 28 | 0 | 0 |
| Megachilidae | *Chelostoma* | *rapunculi* | 0 | 0 | 0 | 0 | 0 | 0 | 0 | 0 | 0 | 1 | 0 | 0 |
| Megachilidae | *Heriades* | *truncorum* | 0 | 0 | 0 | 0 | 1 | 0 | 0 | 0 | 0 | 0 | 0 | 0 |
| Megachilidae | *Megachile* | *ericetorum* | 0 | 0 | 0 | 0 | 0 | 1 | 0 | 0 | 0 | 0 | 0 | 0 |
| Megachilidae | *Megachile* | *maritima* | 0 | 0 | 0 | 0 | 1 | 0 | 0 | 0 | 0 | 0 | 0 | 0 |
| Megachilidae | *Megachile* | *willughbiella* | 0 | 0 | 0 | 0 | 0 | 0 | 0 | 0 | 0 | 1 | 1 | 0 |
| Megachilidae | *Osmia* | *bicornis* | 1 | 0 | 0 | 0 | 0 | 0 | 0 | 0 | 0 | 0 | 0 | 1 |
| Melittidae | *Dasypoda* | *hirtipes* | 0 | 0 | 1 | 0 | 0 | 1 | 3 | 0 | 0 | 0 | 0 | 0 |
