# Supplementary material for: The proportion of impervious surfaces at the landscape scale structures wild bee assemblages in a densely populated region
Source: Ecol Evol. 2016 Aug 25;6(18):6599–615. doi: 10.1002/ece3.2374 (PMC5058531; doi:10.1002/ece3.2374)
Supplement: Supplementary file 2 — Table 2‐1,‐2,‐3,‐4. Species distribution of flowering plant species in each experimental site. [file ECE3-6-6599-s002.docx]

**Table S2-1**: Species distribution of flowering plant species in each experimental site

|  |  | site 1 | site 2 | site 3 | site 4 | site 5 | site 6 | site 7 | site 8 | site 9 | site 10 | site 11 | site 12 |
| --- | --- | --- | --- | --- | --- | --- | --- | --- | --- | --- | --- | --- | --- |
| Familly | Species |  |  |  |  |  |  |  |  |  |  |  |  |
| Adoxaceae | *Sambucus nigra* | 0 | 0 | 0 | 5 | 0 | 0 | 0 | 0 | 0 | 0 | 0 | 0 |
| Adoxaceae | *Viburnum tinus* | 0 | 0 | 0 | 0 | 0 | 0 | 0 | 0 | 0 | 0 | 0 | 1 |
| Apiaceae | *Aegopodium podagraria* | 0 | 0 | 0 | 0 | 3 | 0 | 0 | 0 | 0 | 0 | 0 | 0 |
| Apiaceae | *Angelica sylvestris* | 1 | 0 | 0 | 0 | 0 | 0 | 0 | 0 | 0 | 2 | 0 | 0 |
| Apiaceae | *Anthriscus sylvestris* | 0 | 0 | 3 | 5 | 8 | 0 | 0 | 0 | 0 | 2 | 0 | 0 |
| Apiaceae | *Chaerophyllum temulum* | 0 | 0 | 0 | 0 | 0 | 0 | 0 | 0 | 2 | 5 | 0 | 0 |
| Apiaceae | *Daucus carota* | 0 | 0 | 12 | 19 | 0 | 0 | 0 | 0 | 0 | 4 | 0 | 0 |
| Apiaceae | *Eryngium campestre* | 0 | 8 | 0 | 0 | 0 | 0 | 0 | 0 | 0 | 0 | 0 | 0 |
| Apiaceae | *Heracleum sphondylium* | 9 | 0 | 7 | 0 | 2 | 3 | 0 | 0 | 0 | 0 | 0 | 0 |
| Apiaceae | *Oenanthe aquatica* | 0 | 0 | 0 | 0 | 0 | 0 | 0 | 0 | 0 | 1 | 0 | 0 |
| Apiaceae | *Pastinaca sativa* | 0 | 0 | 1 | 0 | 0 | 0 | 0 | 0 | 0 | 0 | 0 | 0 |
| Apiaceae | *Seseli anuum* | 0 | 0 | 0 | 0 | 0 | 0 | 0 | 0 | 0 | 2 | 0 | 0 |
| Araceae | *Arum italicum* | 0 | 0 | 0 | 0 | 0 | 0 | 0 | 0 | 6 | 0 | 0 | 0 |
| Araceae | *Arum maculatum* | 1 | 0 | 0 | 0 | 1 | 0 | 0 | 0 | 0 | 0 | 0 | 0 |
| Araliaceae | *Hedera helix* | 10 | 0 | 1 | 11 | 12 | 0 | 2 | 0 | 9 | 8 | 0 | 1 |
| Asparagaceae | *Hyacinthus orientalis* | 0 | 0 | 0 | 0 | 0 | 0 | 0 | 0 | 0 | 0 | 0 | 2 |
| Asparagaceae | *Polygonatum multiflorum* | 3 | 0 | 0 | 5 | 0 | 0 | 0 | 0 | 0 | 0 | 0 | 0 |
| Asteraceae | *Achillea millefolium* | 0 | 9 | 0 | 0 | 8 | 0 | 11 | 0 | 2 | 6 | 0 | 0 |
| Asteraceae | *Artemisia campestris* | 0 | 0 | 0 | 3 | 0 | 0 | 0 | 0 | 0 | 0 | 0 | 0 |
| Asteraceae | *Artemisia vulgaris* | 0 | 0 | 2 | 0 | 0 | 1 | 0 | 0 | 0 | 0 | 5 | 0 |
| Asteraceae | *Aster lanceolatus* | 0 | 0 | 0 | 0 | 0 | 0 | 0 | 0 | 0 | 4 | 0 | 0 |
| Asteraceae | *Bellis perennis* | 1 | 0 | 0 | 0 | 0 | 0 | 15 | 0 | 12 | 1 | 10 | 0 |
| Asteraceae | *Calendula officinalis* | 0 | 0 | 0 | 0 | 0 | 0 | 0 | 0 | 0 | 0 | 2 | 0 |
| Asteraceae | *Centaurea jacea* | 0 | 6 | 0 | 15 | 0 | 0 | 0 | 0 | 0 | 0 | 0 | 0 |
| Asteraceae | *Cirsium arvense* | 0 | 1 | 3 | 0 | 0 | 4 | 0 | 2 | 0 | 4 | 7 | 0 |
| Asteraceae | *Cirsium palustre* | 1 | 0 | 0 | 0 | 0 | 0 | 0 | 0 | 0 | 0 | 0 | 0 |
| Asteraceae | *Cirsium vulgare* | 0 | 0 | 3 | 0 | 1 | 0 | 0 | 2 | 0 | 2 | 0 | 0 |
| Asteraceae | *Crepis capillaris* | 0 | 0 | 0 | 3 | 1 | 0 | 0 | 5 | 3 | 0 | 4 | 0 |
| Asteraceae | *Galinsoga quadriradiata* | 0 | 0 | 0 | 0 | 0 | 0 | 0 | 1 | 0 | 0 | 7 | 0 |
| Asteraceae | *Gnaphalium uliginosum* | 0 | 0 | 0 | 1 | 0 | 0 | 0 | 0 | 0 | 0 | 0 | 0 |
| Asteraceae | *Hieracium murorum* | 0 | 0 | 0 | 2 | 0 | 0 | 0 | 0 | 0 | 0 | 0 | 0 |
| Asteraceae | *Hypochaeris radicata* | 1 | 0 | 0 | 9 | 0 | 0 | 14 | 11 | 0 | 1 | 0 | 0 |
| Asteraceae | *Lapsana communis* | 0 | 0 | 0 | 0 | 3 | 0 | 0 | 0 | 0 | 1 | 0 | 0 |
| Asteraceae | *Leucanthemum vulgare* | 0 | 1 | 0 | 0 | 0 | 0 | 0 | 0 | 0 | 0 | 0 | 0 |
| Asteraceae | *Matricaria discoidea* | 0 | 0 | 0 | 0 | 0 | 1 | 0 | 0 | 0 | 0 | 0 | 0 |
| Asteraceae | *Matricaria perforata* | 0 | 0 | 1 | 0 | 0 | 0 | 0 | 0 | 0 | 1 | 3 | 0 |
| Asteraceae | *Matricaria recutita* | 0 | 7 | 0 | 0 | 0 | 0 | 0 | 0 | 0 | 0 | 0 | 0 |
| Asteraceae | *Pulicaria dysenterica* | 10 | 0 | 0 | 0 | 0 | 0 | 0 | 0 | 0 | 0 | 0 | 0 |
| Asteraceae | *Senecio erucifolius* | 0 | 0 | 2 | 0 | 0 | 0 | 0 | 0 | 0 | 0 | 0 | 0 |
| Asteraceae | *Senecio jacobaea* | 0 | 0 | 0 | 13 | 0 | 0 | 2 | 0 | 1 | 6 | 0 | 0 |
| Asteraceae | *Senecio ovatus* | 0 | 0 | 0 | 2 | 0 | 0 | 0 | 0 | 0 | 0 | 0 | 0 |
| Asteraceae | *Senecio vulgare* | 0 | 0 | 0 | 0 | 0 | 0 | 2 | 12 | 0 | 1 | 1 | 0 |
| Asteraceae | *Sonchus asper* | 0 | 1 | 0 | 0 | 1 | 3 | 2 | 15 | 0 | 3 | 4 | 0 |
| Asteraceae | *Sonchus oleraceus* | 0 | 0 | 1 | 0 | 0 | 0 | 0 | 0 | 1 | 3 | 0 | 0 |
| Asteraceae | *Taraxacum ruderale* | 10 | 10 | 16 | 1 | 4 | 0 | 8 | 9 | 5 | 0 | 11 | 0 |
| Balsaminaceae | *Impatiens parviflora* | 0 | 0 | 0 | 0 | 0 | 0 | 0 | 0 | 0 | 2 | 0 | 0 |
| Berberidaceae | *Mahonia aquifolium* | 0 | 0 | 0 | 0 | 0 | 0 | 3 | 0 | 7 | 0 | 0 | 0 |
| Boraginaceae | *Myosotis arvensis* | 0 | 0 | 0 | 14 | 0 | 0 | 1 | 0 | 0 | 1 | 0 | 0 |

**Table S2-2**: Species distribution of flowering plant species in each experimental site

|  |  | site 1 | site 2 | site 3 | site 4 | site 5 | site 6 | site 7 | site 8 | site 9 | site 10 | site 11 | site 12 |  |
| --- | --- | --- | --- | --- | --- | --- | --- | --- | --- | --- | --- | --- | --- | --- |
| Familly | Species |  |  |  |  |  |  |  |  |  |  |  |  |  |
| Brassicaceae | *Alliaria petiolata* | 0 | 0 | 0 | 10 | 2 | 0 | 0 | 0 | 4 | 0 | 0 | 0 |  |
| Brassicaceae | *Arabidopsis thaliana* | 0 | 0 | 0 | 0 | 0 | 0 | 0 | 1 | 0 | 0 | 0 | 0 |  |
| Brassicaceae | *Capsella bursa-pastoris* | 0 | 0 | 0 | 0 | 0 | 0 | 0 | 0 | 4 | 2 | 10 | 0 |  |
| Brassicaceae | *Cardamine hirsuta* | 0 | 0 | 0 | 3 | 0 | 0 | 4 | 10 | 3 | 2 | 3 | 0 |  |
| Brassicaceae | *Cardamine pratensis* | 5 | 0 | 0 | 0 | 0 | 0 | 0 | 0 | 0 | 0 | 0 | 0 |  |
| Brassicaceae | *Coronopus didymus* | 0 | 0 | 0 | 0 | 0 | 0 | 0 | 0 | 0 | 2 | 0 | 0 |  |
| Brassicaceae | *Erophila verna* | 0 | 0 | 0 | 0 | 0 | 0 | 2 | 3 | 2 | 0 | 0 | 0 |  |
| Brassicaceae | *Sinapis arvensis* | 0 | 0 | 3 | 0 | 2 | 2 | 0 | 0 | 0 | 1 | 0 | 0 |  |
| Brassicaceae | *Sisymbrium officinale* | 0 | 0 | 0 | 0 | 0 | 0 | 0 | 0 | 0 | 0 | 8 | 0 |  |
| Campanulaceae | *Campanula trachelium* | 0 | 0 | 0 | 0 | 0 | 0 | 0 | 0 | 0 | 2 | 0 | 0 |  |
| Caprifoliaceae | *Abelia grandiflora* | 0 | 0 | 0 | 0 | 0 | 0 | 0 | 0 | 0 | 0 | 0 | 1 |  |
| Caprifoliaceae | *Lonicera periclymenum* | 0 | 0 | 0 | 1 | 0 | 0 | 0 | 0 | 0 | 0 | 0 | 0 |  |
| Caprifoliaceae | *Lonicera xylosteum* | 0 | 0 | 0 | 0 | 0 | 0 | 0 | 0 | 5 | 0 | 0 | 0 |  |
| Caryophyllaceae | *Arenaria serpyllifolia* | 0 | 0 | 0 | 0 | 0 | 0 | 0 | 7 | 0 | 2 | 1 | 0 |  |
| Caryophyllaceae | *Cerastium fontanum* | 3 | 2 | 4 | 16 | 0 | 0 | 15 | 4 | 12 | 6 | 4 | 0 |  |
| Caryophyllaceae | *Cerastium glomeratum* | 0 | 0 | 0 | 0 | 0 | 0 | 0 | 2 | 0 | 0 | 0 | 0 |  |
| Caryophyllaceae | *Moehringia trinervia* | 0 | 0 | 0 | 1 | 0 | 0 | 0 | 0 | 0 | 0 | 0 | 0 |  |
| Caryophyllaceae | *Sagina apetala* | 0 | 0 | 0 | 0 | 0 | 0 | 0 | 0 | 0 | 1 | 0 | 0 |  |
| Caryophyllaceae | *Sagina procumbens* | 0 | 0 | 0 | 0 | 0 | 0 | 0 | 8 | 0 | 0 | 0 | 0 |  |
| Caryophyllaceae | *Silene latifolia subsp. alba* | 0 | 0 | 1 | 0 | 0 | 0 | 0 | 0 | 10 | 0 | 2 | 0 |  |
| Caryophyllaceae | *Silene vulgaris* | 0 | 0 | 2 | 9 | 0 | 0 | 0 | 0 | 3 | 0 | 0 | 0 |  |
| Caryophyllaceae | *Stellaria graminea* | 15 | 0 | 0 | 0 | 0 | 0 | 0 | 0 | 0 | 0 | 0 | 0 |  |
| Caryophyllaceae | *Stellaria media* | 0 | 0 | 0 | 0 | 0 | 0 | 0 | 2 | 5 | 2 | 1 | 0 |  |
| Celastraceae | *Evonymus europaeus* | 2 | 0 | 0 | 5 | 0 | 0 | 0 | 0 | 3 | 3 | 0 | 0 |  |
| Convolvulaceae | *Calystegia sepium* | 0 | 0 | 0 | 0 | 0 | 0 | 0 | 1 | 0 | 4 | 3 | 0 |  |
| Convolvulaceae | *Convolvulus arvensis* | 10 | 4 | 7 | 0 | 1 | 0 | 1 | 0 | 0 | 1 | 2 | 0 |  |
| Cornaceae | *Cornus mas* | 0 | 0 | 0 | 0 | 0 | 0 | 0 | 0 | 6 | 0 | 0 | 0 |  |
| Crassulaceae | *Sedum acre* | 0 | 0 | 0 | 0 | 0 | 0 | 0 | 5 | 0 | 0 | 0 | 0 |  |
| Dipsacaceae | *Dipsacus fullonum* | 0 | 0 | 0 | 0 | 0 | 0 | 0 | 0 | 0 | 1 | 0 | 0 |  |
| Ericaceae | *Calluna vulgaris* | 0 | 0 | 0 | 0 | 0 | 0 | 0 | 1 | 0 | 0 | 0 | 0 |  |
| Ericaceae | *Rhododenron azaleatsrum* | 0 | 0 | 0 | 0 | 0 | 0 | 0 | 0 | 0 | 3 | 0 | 0 |  |
| Ericaceae | *Rhododenron sp.* | 0 | 0 | 0 | 0 | 0 | 0 | 0 | 0 | 0 | 6 | 0 | 0 |  |
| Euphorbiaceae | *Euphorbia helioscopia* | 0 | 0 | 0 | 0 | 0 | 0 | 0 | 2 | 0 | 8 | 10 | 0 |  |
| Fabaceae | *Cytisus scoparius* | 0 | 0 | 0 | 26 | 0 | 0 | 0 | 0 | 0 | 1 | 0 | 0 |  |
| Fabaceae | *Lathyrus latifolius* | 0 | 0 | 0 | 0 | 0 | 0 | 2 | 0 | 0 | 0 | 0 | 0 |  |
| Fabaceae | *Lotus corniculatus* | 0 | 1 | 0 | 0 | 0 | 0 | 0 | 0 | 0 | 0 | 0 | 0 |  |
| Fabaceae | *Lotus pedunculatus* | 8 | 0 | 0 | 0 | 0 | 0 | 0 | 0 | 0 | 0 | 0 | 0 |  |
| Fabaceae | *Medicago arabica* | 0 | 0 | 0 | 0 | 0 | 0 | 0 | 0 | 2 | 0 | 0 | 0 |  |
| Fabaceae | *Medicago lupulina* | 0 | 2 | 3 | 5 | 0 | 0 | 10 | 13 | 9 | 0 | 0 | 0 |  |
| Fabaceae | *Medicago sativa* | 0 | 0 | 1 | 0 | 0 | 0 | 0 | 0 | 0 | 0 | 0 | 0 |  |
| Fabaceae | *Ononis repens* | 0 | 0 | 0 | 0 | 0 | 0 | 0 | 0 | 1 | 0 | 0 | 0 |  |
| Fabaceae | *Ononis spinosa* | 0 | 8 | 0 | 0 | 0 | 0 | 0 | 0 | 0 | 0 | 0 | 0 |  |
| Fabaceae | *Robinia pseudoacacia* | 0 | 0 | 0 | 0 | 0 | 0 | 0 | 0 | 10 | 0 | 8 | 0 |  |
| Fabaceae | *Trifolim pratense* | 2 | 7 | 3 | 0 | 0 | 0 | 0 | 0 | 9 | 1 | 0 | 0 |  |
| Fabaceae | *Trifolium arvense* | 0 | 0 | 0 | 7 | 0 | 0 | 0 | 0 | 0 | 0 | 0 | 0 |  |
| Fabaceae | *Trifolium repens* | 24 | 8 | 3 | 0 | 0 | 0 | 16 | 0 | 0 | 1 | 15 | 0 |  |
| Fabaceae | *Vicia cracca* | 0 | 0 | 0 | 0 | 11 | 0 | 0 | 0 | 0 | 0 | 0 | 0 |  |

**Table S2-3**: Species distribution of flowering plant species in each experimental site

|  |  | site 1 | site 2 | site 3 | site 4 | site 5 | site 6 | site 7 | site 8 | site 9 | site 10 | site 11 | site 12 |
| --- | --- | --- | --- | --- | --- | --- | --- | --- | --- | --- | --- | --- | --- |
| Familly | Species |  |  |  |  |  |  |  |  |  |  |  |  |
| Fabaceae | *Vicia faba var minor* | 0 | 0 | 0 | 0 | 20 | 0 | 0 | 0 | 0 | 0 | 0 | 0 |
| Fabaceae | *Vicia hirsuta* | 0 | 0 | 29 | 14 | 2 | 0 | 9 | 0 | 0 | 4 | 0 | 0 |
| Fabaceae | *Vicia sativa* | 0 | 8 | 0 | 0 | 0 | 0 | 8 | 0 | 0 | 0 | 0 | 0 |
| Fabaceae | *Vicia tetrasperma* | 0 | 0 | 7 | 25 | 0 | 0 | 0 | 0 | 0 | 0 | 0 | 0 |
| Fagaceae | *Castanea sativa* | 0 | 0 | 0 | 0 | 0 | 0 | 0 | 0 | 1 | 0 | 0 | 0 |
| Geraniaceae | *Erodium cicutarium* | 0 | 0 | 0 | 6 | 0 | 0 | 0 | 0 | 0 | 0 | 0 | 0 |
| Geraniaceae | *Geranium dissectum* | 0 | 10 | 10 | 6 | 0 | 0 | 0 | 0 | 0 | 1 | 0 | 0 |
| Geraniaceae | *Geranium lucidum* | 0 | 0 | 0 | 0 | 0 | 0 | 0 | 0 | 1 | 0 | 0 | 0 |
| Geraniaceae | *Geranium molle* | 0 | 0 | 1 | 1 | 2 | 0 | 2 | 0 | 16 | 0 | 3 | 0 |
| Geraniaceae | *Geranium robertianum* | 0 | 0 | 1 | 9 | 4 | 0 | 0 | 0 | 3 | 0 | 0 | 0 |
| Hydrangeaceae | *Hydrangea petiolaris* | 0 | 0 | 0 | 0 | 0 | 0 | 0 | 0 | 0 | 1 | 0 | 0 |
| Hypericaceae | *Hypericum hirsutum* | 0 | 0 | 0 | 0 | 0 | 0 | 0 | 0 | 0 | 1 | 0 | 0 |
| Hypericaceae | *Hypericum perforatum* | 0 | 0 | 0 | 21 | 0 | 0 | 2 | 0 | 0 | 0 | 0 | 0 |
| Lamiaceae | *Ballota nigra* | 0 | 0 | 0 | 0 | 0 | 0 | 0 | 0 | 0 | 0 | 6 | 0 |
| Lamiaceae | *Galeopsis tetrahit* | 0 | 0 | 0 | 4 | 0 | 0 | 0 | 0 | 0 | 0 | 0 | 0 |
| Lamiaceae | *Glechoma hederacea* | 5 | 0 | 0 | 0 | 9 | 0 | 0 | 0 | 0 | 7 | 0 | 0 |
| Lamiaceae | *Lamium album* | 2 | 0 | 0 | 0 | 0 | 0 | 0 | 0 | 0 | 1 | 0 | 0 |
| Lamiaceae | *Lamium amplexicaule* | 0 | 0 | 0 | 0 | 0 | 0 | 0 | 0 | 0 | 1 | 6 | 0 |
| Lamiaceae | *Lamium maculatum* | 0 | 0 | 0 | 0 | 0 | 0 | 0 | 0 | 1 | 3 | 0 | 0 |
| Lamiaceae | *Lamium purpureum* | 0 | 0 | 0 | 4 | 0 | 0 | 0 | 0 | 3 | 0 | 0 | 0 |
| Lamiaceae | *Lycopus europaeus* | 1 | 0 | 0 | 0 | 0 | 0 | 0 | 0 | 0 | 0 | 0 | 0 |
| Lamiaceae | *Mentha aquatica* | 10 | 0 | 0 | 0 | 0 | 0 | 0 | 0 | 0 | 0 | 0 | 0 |
| Lamiaceae | *Mentha suaveolens* | 22 | 0 | 0 | 0 | 0 | 0 | 0 | 0 | 0 | 1 | 0 | 0 |
| Lamiaceae | *Origanum vulgare* | 0 | 0 | 0 | 2 | 0 | 0 | 0 | 0 | 0 | 0 | 0 | 0 |
| Lamiaceae | *Prunella vulgaris* | 2 | 11 | 0 | 0 | 0 | 0 | 0 | 0 | 0 | 1 | 0 | 0 |
| Lamiaceae | *Stachys sylvatica* | 6 | 1 | 0 | 0 | 3 | 0 | 0 | 0 | 0 | 0 | 0 | 0 |
| Liliaceae | *Convallaria majalis* | 0 | 0 | 0 | 0 | 0 | 0 | 0 | 0 | 10 | 0 | 0 | 0 |
| Malvaceae | *Alcea rosea* | 0 | 0 | 0 | 0 | 0 | 0 | 0 | 0 | 0 | 1 | 0 | 0 |
| Malvaceae | *Althea syriaca* | 0 | 0 | 0 | 0 | 0 | 0 | 0 | 0 | 0 | 0 | 0 | 1 |
| Malvaceae | *Malva neglecta* | 0 | 0 | 0 | 0 | 0 | 0 | 0 | 0 | 0 | 1 | 1 | 0 |
| Myrsinaceae | *Anagallis arvensis* | 0 | 0 | 2 | 0 | 0 | 3 | 0 | 0 | 0 | 0 | 3 | 0 |
| Oleaceae | *Ligustrum vulgare* | 0 | 0 | 0 | 0 | 0 | 0 | 0 | 0 | 2 | 0 | 0 | 0 |
| Onagraceae | *Epilobium ciliatum* | 0 | 0 | 0 | 0 | 0 | 0 | 0 | 0 | 2 | 0 | 0 | 0 |
| Onagraceae | *Epilobium hirsutum* | 1 | 0 | 0 | 0 | 0 | 0 | 0 | 0 | 0 | 0 | 0 | 0 |
| Onagraceae | *Epilobium tetragonum* | 0 | 0 | 4 | 0 | 0 | 0 | 0 | 0 | 0 | 0 | 0 | 0 |
| Orchidaceae | *Listera ovata* | 0 | 0 | 0 | 0 | 3 | 0 | 0 | 0 | 0 | 0 | 0 | 0 |
| Oxalidaceae | *Oxalis corniculata* | 0 | 0 | 0 | 0 | 0 | 0 | 0 | 0 | 0 | 3 | 0 | 0 |
| Papaveraceae | *Chelidonium majus* | 0 | 0 | 0 | 0 | 0 | 0 | 0 | 0 | 0 | 3 | 0 | 0 |
| Papaveraceae | *Escholtzia californica* | 0 | 0 | 0 | 0 | 0 | 0 | 0 | 4 | 0 | 0 | 0 | 0 |
| Papaveraceae | *Fumaria officinalis* | 0 | 0 | 0 | 0 | 0 | 5 | 0 | 0 | 0 | 0 | 0 | 0 |
| Papaveraceae | *Papaver rhoeas* | 0 | 0 | 1 | 0 | 0 | 1 | 0 | 2 | 1 | 0 | 0 | 0 |
| Plantaginaceae | *Linaria vulgaris* | 0 | 0 | 0 | 6 | 0 | 0 | 0 | 0 | 0 | 0 | 0 | 0 |
| Plantaginaceae | *Veronica arvensis* | 0 | 0 | 2 | 13 | 0 | 0 | 13 | 4 | 10 | 4 | 5 | 0 |
| Plantaginaceae | *Veronica chamaedrys* | 10 | 0 | 0 | 0 | 11 | 0 | 0 | 0 | 0 | 0 | 0 | 0 |
| Plantaginaceae | *Veronica hederifolia* | 0 | 0 | 0 | 0 | 9 | 0 | 0 | 0 | 5 | 0 | 0 | 0 |
| Plantaginaceae | *Veronica persica* | 0 | 0 | 3 | 0 | 0 | 0 | 0 | 0 | 4 | 0 | 10 | 0 |
| Polygonaceae | *Fallopia convolvulus* | 0 | 0 | 1 | 0 | 2 | 16 | 0 | 0 | 1 | 0 | 0 | 0 |
| Polygonaceae | *Fallopia dumetorum* | 0 | 0 | 0 | 2 | 0 | 0 | 0 | 0 | 0 | 0 | 0 | 0 |

**Table S2-4**: Species distribution of flowering plant species in each experimental site

|  |  | site 1 | site 2 | site 3 | site 4 | site 5 | site 6 | site 7 | site 8 | site 9 | site 10 | site 11 | site 12 |
| --- | --- | --- | --- | --- | --- | --- | --- | --- | --- | --- | --- | --- | --- |
| Familly | Species |  |  |  |  |  |  |  |  |  |  |  |  |
| Polygonaceae | *Polygonum aviculare* | 0 | 0 | 0 | 0 | 0 | 1 | 0 | 0 | 0 | 0 | 0 | 0 |
| Polygonaceae | *Polygonum aviculare* | 0 | 10 | 2 | 0 | 0 | 0 | 0 | 2 | 0 | 0 | 1 | 0 |
| Polygonaceae | *Polygonum hydropiper* | 1 | 0 | 0 | 0 | 0 | 0 | 0 | 0 | 0 | 0 | 0 | 0 |
| Polygonaceae | *Polygonum persicaria* | 0 | 0 | 0 | 0 | 2 | 0 | 0 | 8 | 0 | 0 | 0 | 0 |
| Primulaceae | *Lysimachia nummularia* | 4 | 0 | 0 | 0 | 9 | 0 | 0 | 0 | 0 | 0 | 0 | 0 |
| Primulaceae | *Primula veris* | 0 | 0 | 0 | 0 | 0 | 0 | 0 | 0 | 0 | 1 | 0 | 0 |
| Ranunculaceae | *Anemone nemorosa* | 6 | 0 | 0 | 0 | 0 | 0 | 0 | 0 | 0 | 0 | 0 | 0 |
| Ranunculaceae | *Ranunculus acris* | 32 | 2 | 0 | 0 | 2 | 0 | 0 | 0 | 0 | 1 | 0 | 0 |
| Ranunculaceae | *Ranunculus bulbosus* | 0 | 4 | 0 | 0 | 0 | 0 | 0 | 0 | 0 | 0 | 0 | 0 |
| Ranunculaceae | *Ranunculus fiicaria* | 0 | 0 | 0 | 0 | 6 | 0 | 0 | 0 | 0 | 0 | 0 | 0 |
| Ranunculaceae | *Ranunculus repens* | 10 | 9 | 0 | 2 | 0 | 0 | 2 | 0 | 0 | 3 | 0 | 0 |
| Resedaceae | *Reseda lutea* | 0 | 0 | 0 | 0 | 0 | 0 | 0 | 0 | 0 | 1 | 0 | 0 |
| Rhamnaceae | *Rhamnus cathartica* | 0 | 0 | 0 | 0 | 0 | 0 | 0 | 0 | 4 | 0 | 0 | 0 |
| Rosaceae | *Agrimonia eupatoria* | 0 | 0 | 2 | 0 | 0 | 0 | 0 | 0 | 0 | 0 | 0 | 0 |
| Rosaceae | *Aphanes arvensis* | 0 | 0 | 0 | 9 | 0 | 0 | 0 | 0 | 0 | 0 | 0 | 0 |
| Rosaceae | *Crataegus monogyna* | 0 | 0 | 1 | 8 | 0 | 0 | 3 | 0 | 0 | 0 | 0 | 0 |
| Rosaceae | *Duchesnea indica* | 0 | 0 | 0 | 0 | 0 | 0 | 0 | 0 | 0 | 2 | 0 | 0 |
| Rosaceae | *Fragaria vesca* | 0 | 0 | 2 | 0 | 0 | 0 | 0 | 0 | 0 | 0 | 0 | 0 |
| Rosaceae | *Geum urbanum* | 1 | 1 | 0 | 10 | 6 | 0 | 0 | 0 | 2 | 1 | 0 | 5 |
| Rosaceae | *Malus domestica* | 0 | 0 | 0 | 0 | 0 | 0 | 0 | 0 | 0 | 0 | 5 | 0 |
| Rosaceae | *Malus sylvestris* | 0 | 0 | 0 | 9 | 0 | 0 | 0 | 0 | 0 | 0 | 0 | 0 |
| Rosaceae | *Picris echioides* | 0 | 0 | 0 | 0 | 2 | 0 | 0 | 12 | 0 | 5 | 3 | 0 |
| Rosaceae | *Picris hieracioides* | 0 | 1 | 11 | 16 | 0 | 9 | 0 | 0 | 0 | 6 | 6 | 0 |
| Rosaceae | *Potentilla anserina* | 20 | 0 | 0 | 0 | 0 | 0 | 0 | 0 | 0 | 0 | 0 | 0 |
| Rosaceae | *Potentilla neumanniana* | 0 | 0 | 0 | 0 | 0 | 0 | 0 | 0 | 6 | 0 | 0 | 0 |
| Rosaceae | *Potentilla reptans* | 0 | 5 | 14 | 0 | 10 | 0 | 0 | 0 | 1 | 5 | 0 | 0 |
| Rosaceae | *Prunus avium* | 0 | 0 | 0 | 3 | 0 | 0 | 0 | 0 | 0 | 0 | 0 | 0 |
| Rosaceae | *Prunus spinosa* | 0 | 0 | 2 | 39 | 0 | 0 | 0 | 0 | 0 | 0 | 0 | 0 |
| Rosaceae | *Rosa arvensis* | 0 | 0 | 0 | 0 | 1 | 0 | 0 | 0 | 0 | 0 | 0 | 0 |
| Rosaceae | *Rosa canina* | 0 | 0 | 2 | 5 | 0 | 0 | 0 | 0 | 0 | 0 | 0 | 0 |
| Rosaceae | *Rubus fruticosus* | 10 | 10 | 9 | 0 | 0 | 0 | 0 | 0 | 0 | 0 | 0 | 0 |
| Rosaceae | *Rubus ulmifolius* | 0 | 0 | 0 | 15 | 0 | 0 | 0 | 0 | 0 | 0 | 0 | 0 |
| Rosaceae | *Sanguisorba minor* | 0 | 9 | 0 | 0 | 0 | 0 | 0 | 0 | 0 | 0 | 0 | 0 |
| Rosaceae | *Spiraea vanhouttei* | 0 | 0 | 0 | 0 | 0 | 0 | 2 | 0 | 0 | 0 | 0 | 0 |
| Rubiaceae | *Galium aparine* | 10 | 0 | 6 | 30 | 9 | 4 | 0 | 0 | 4 | 0 | 0 | 0 |
| Rubiaceae | *Galium mollugo* | 0 | 0 | 0 | 0 | 0 | 0 | 0 | 0 | 0 | 3 | 0 | 0 |
| Rutaceae | *Choisya ternata* | 0 | 0 | 0 | 0 | 0 | 0 | 0 | 0 | 0 | 0 | 0 | 4 |
| Salicaceae | *Salix sp.* | 0 | 0 | 0 | 0 | 10 | 0 | 0 | 0 | 0 | 0 | 0 | 0 |
| Sapindaceae | *Acer campestre* | 0 | 0 | 0 | 0 | 0 | 0 | 0 | 0 | 0 | 2 | 0 | 0 |
| Sapindaceae | *Acer platanoides* | 4 | 0 | 0 | 0 | 11 | 0 | 0 | 0 | 7 | 0 | 0 | 0 |
| Sapindaceae | *Acer pseudoplatanus* | 0 | 0 | 0 | 0 | 17 | 0 | 0 | 10 | 1 | 7 | 0 | 0 |
| Sapindaceae | *Aesculus hippocastanum* | 0 | 0 | 0 | 0 | 10 | 0 | 0 | 0 | 0 | 0 | 1 | 8 |
| Saxifragaceae | *Saxifraga tridactylites* | 0 | 0 | 0 | 0 | 0 | 0 | 0 | 2 | 0 | 0 | 0 | 0 |
| Scrophulariaceae | *Verbascum thapsus* | 0 | 0 | 0 | 0 | 0 | 0 | 0 | 0 | 0 | 2 | 0 | 0 |
| Solanaceae | *Solanum nigrum* | 0 | 0 | 1 | 0 | 1 | 0 | 0 | 9 | 0 | 1 | 1 | 0 |
| Tiliaceae | *Tilia platyphyllos* | 0 | 0 | 0 | 0 | 20 | 0 | 0 | 0 | 0 | 0 | 0 | 6 |
| Valerianaceae | *Valerianella locusta* | 0 | 0 | 4 | 22 | 0 | 0 | 0 | 2 | 0 | 0 | 0 | 0 |
| Verbenaceae | *Verbena officinalis* | 0 | 0 | 0 | 0 | 0 | 0 | 0 | 0 | 10 | 0 | 0 | 0 |
| violaceae | *Viola arvensis* | 0 | 0 | 1 | 0 | 0 | 0 | 0 | 0 | 0 | 0 | 0 | 0 |
| violaceae | *Viola odorata* | 0 | 3 | 0 | 0 | 3 | 0 | 0 | 1 | 0 | 0 | 0 | 0 |
| violaceae | *Viola reichenbachiana* | 0 | 0 | 0 | 8 | 0 | 0 | 0 | 0 | 0 | 0 | 0 | 0 |
| Xanthorrhoeaceae | *Hemerocallis fulva* | 0 | 0 | 0 | 0 | 0 | 0 | 0 | 0 | 3 | 0 | 0 | 0 |
